# Lipid and renal profile in assessing the severity of alcoholic liver disease

**DOI:** 10.6026/973206300181036

**Published:** 2022-10-31

**Authors:** Chitrasivasankari G, Gomathi V, Nachiappan R, Gurumoorthy Kaarthikeyan, Mahalakshmi S

**Affiliations:** 1 Institute of Biochemistry, Madras Medical College, RGGGH, Chennai, India; 2 Saveetha Dental College & Hospital, Saveetha Institute of Medical and Technical Sciences (SIMATS), Chennai - 77, India; 3 Department of Biochemistry, Madurai Medical College, Madurai, Tamilnadu, India

**Keywords:** Alcoholic liver disease, lipid profile, hepato-renal syndrome, health

## Abstract

Lipid and Renal dysfunction in Alcoholic liver disease (ALD) patients occurs either due to multi-organ involvement or secondary to alcoholism. This study was conducted to evaluate the role of lipid and renal parameters in assessing the severity of progression of ALD. Sixty cases of ALD (two groups based on compensated and decompensated features) and thirty healthy controls for comparison were included. Lipid profile (Total Cholesterol, LDL, HDL and Triglycerides) and renal parameters (serum urea, creatinine and uric acid), total and direct bilirubin, total protein and albumin were measured using automated chemistry analyzer. There was a significant decrease in Total cholesterol ,LDL and HDL levels and increased triglycerides when compared to controls (mean of 128.4 ± 59 vs 155 ± 27.2, 77 ± 44.3 vs 97.4 ± 27.2, 28.3 ± 18 vs 39.5 ± 14.1 and 115.8 ± 70.4 vs 91 ± 38 mg/dL respectively). Lipid profile showed a linear decrease while progressing from compensated to decompensated ALD. Renal parameters revealed a statistically significant decrease in serum urea ,increased creatinine and uric acid levels when compared to controls (17.57±2.96 vs23.73±4.94, 1.12±0.55 vs0.88±0.16,6.60±1.32 vs 4.68±1.40 mg/dL respectively).Total cholesterol and HDL showed a linear decrease when ALD progresses. Serum uric acid showed an early increase in compensated stage of ALD. This study inferred that Total cholesterol, TGL, HDL and uric acid can be used for assessing the severity of progression of ALD.

## Background:

Globally, alcoholism is one among the common etiologies of liver disease. Simple steatosis occurs in alcoholic liver injury in early stages later progresses to hepatitis, fibrosis and eventually ends up in cirrhosis and hepatocellular carcinoma [1-2]. Alcoholic fatty liver progresses to cirrhosis in 30-35% of patients [3]. Obesity, genetic factors, chronic alcohol consumption and viral hepatitis are the common risk factors for alcoholic liver disease (ALD) [4]. Alcohol induced liver injury depends on the amount of alcohol consumed, duration and drinking pattern. Alcohol intake leads to 50% of deaths due to hepatic etiology in males of age group more than 15 years [5]. Fourteenth common cause of death globally is cirrhosis and fourth common cause of death in Europe [6]. Most of the alcoholic liver disease patients have their life span till sixth decade of life. CHILD PUGH score is used to assess the severity, prognosis and selection of patients for liver resection in hepatocellular carcinoma in chronic liver disease patients. In ALD, renal dysfunction is common. Renal involvement occurs when multiple organs are affected in ALD or in advanced stages of alcoholic liver disease. Acute kidney failure and chronic kidney disease are common presentations in alcoholic liver disease [7]. Hepatorenal syndrome is a unique presentation which occurs in chronic liver disease of alcoholic etiology. The two phases of cirrhosis are the compensated phase, also known as the asymptomatic phase, and the decompensated phase, also known as the progressing phase. Except for oesophageal varices, patients will be generally asyptomatic during the compensated period. They will experience the typical complaints, such as nausea without dyspnea, stomach pain and swelling, and vomiting [8]. Portal vein pressure will be normal. Only in the latter half of this phase do the majority of alcoholics with liver damage become apparent [9]. This stage can be reversed, and the outlook is improved. Through dietary shortages, metabolic irregularities, altered renal function, bleeding varices, yellowish discoloration of the sclera and mucous membranes, and portosystemic encephalopathy, the condition will proceed to a decompensated stage. When combined with hepato-renal syndrome, spontaneous bacterial peritonitis, hepato-pulmonary syndrome, and hepatocellular malignancy, this irreversible stage has a poor prognosis [10].

## Materials & Methods:

This study was conducted in the Institute of Biochemistry and Medical gastroenterology in Madras Medical College and Rajiv Gandhi Government General Hospital, Chennai. Ninety human subjects were included in the study. Controls were selected from the outpatient department during their visit to hospital for non hepatic etiology. Apparently healthy individuals, thirty in number with normal ultrasound and total abstinence from alcohol were considered as controls for the study. Cases, sixty in number were selected from Gastro enterology department, Rajiv Gandhi Government General Hospital, Chennai. Cases include sixty patients with ALD based on ultrasound findings. They were selected only after getting informed consent from the patient and divided into two groups based on compensated and decompensated features. GROUP 1 includes thirty ALD patients with compensated features (varices may or may not be present but no ascites) GROUP 2 includes thirty ALD patients with jaundice, ascites and hepatic encephalopathy. Group-3 includes thirty age and sex matched healthy individuals were included as controls. Inclusion criteria include patients with ALD diagnosed by ultrasound or liver biopsy. Patients with non-alcoholic liver disease, viral hepatitis, Autoimmune liver disease, Genetic or metabolic liver disease like Wilson’s disease and alpha 1 antitrypsin deficiency, inflammatory conditions like ulcerative colitis, Crohns disease, and pneumonia and Hepatocellular carcinoma are excluded. Fasting venous blood about 5mL was drawn from antecubital vein under aseptic precautions. Serum separated by centrifugation at 3000 rpm for 15 minutes. Biochemical investigations include total bilirubin(TB) ,direct bilirubin(DB) , alkaline phosphatase (ALP), total protein , albumin ,total cholesterol(TC) , triglycerides(TGL) ,LDL cholesterol ,HDL cholesterol, urea, creatinine and uric acid. These parameters were done in patients with ALD and healthy controls using a conventional automated chemistry analyzer. Diazo Method of Pearlman and Lee, an endpoint method is used for estimation of total and direct bilirubin. Serum ALP was estimated by IFCC-Kinetic Method. Total protein was estimated by Biuret method, an End point assay. Bromocresol green, an endpoint assay is used for estimation of serum albumin. Total cholesterol estimation was done by Cholesterol Esterase-Cholesterol Oxidase method. Plasma triglycerides were estimated by enzymatic method, End point by colorimetry. HDL Cholesterol was estimated by Phosphotungstic acid method, End point assay. LDL Cholesterol was calculated by Friedewald’s formula. Urea estimation was done by Enzymatic Urease-Glutamate Dehydrogenase Kinetic method, creatinine by modified jaffe's kinetic method and uric acid by uricase method.

## Results:

The statistical data were analyzed using SPSS (Statistical Package for Social Science) version 16.0 software. Patients with ALD and healthy controls are tested for a number of analytes that reflect the functioning of the liver, such as TB, DB, ALP, total protein, albumin, TC, TGL, HDL cholesterol, LDL cholesterol, urea, and uric acid. For each study group, the mean and standard deviation were computed. To compare the mean values, the students’t test was used. The p value was determined. A p value of 0.05 or less is regarded as significant. Analysis of Variation was used to examine various groups of ALD with varying lipid and renal profile components (ANOVA TEST). In a group with healthy controls, this test was used to compare hepatic and renal parameters between compensated and decompensated patients of ALD. The study included sixty patients with ALD and thirty apparently healthy controls. The mean age of the cases was 45 years and that of controls was 41 years. Lipid profile parameters were analyzed. TC, HDLc and LDLc were low and TGL levels are high in cases when compared to controls (Table 1 - see PDF). The mean value of TC in cases was 128.4 ± 59mg/dL which is low when compared to controls 155 ± 27.2mg/dL and p Value found to be significant. Similarly, mean LDLc and HDLc levels are also low when compared to controls (77 ± 44.3 vs 97.4 ± 27.2mg/dL and 28.3 ± 18 vs 39.5 ± 14.1mg/dL) as in Table 1(see PDF). In contrast, cases in table 1 showed an increased mean triglyceride levels when compared to the healthy control group (115.8 ± 70.4 vs 91 ± 38 mg/dL) with significant p Value. The ALD patients were divided into two groups based on compensated and decompensated features. Table 2 reveals a significant change in TC levels between controls and decompensated ALD (155.13+27.25mg/dL and139.67+34.92 mg/dL). Serum TGL levels are also markedly elevated in compensated and decompensated cases when compared to controls (146.43+31.48mg/dL, 164.50+48.93 mg/dL and 91.07+38.45mg/dL) and has significant p value.LDL levels does not show any marked changes but HDL levels show significant decrease in decompensated ALD when compared to controls (21.67+9.7mg/dL and 39.57+14.18 mg/dL). Hence TC, TGL and HDLc levels can be used as parameters for assessment of severity of ALD. Among the renal parameters, as in Table 1(see PDF) Urea levels show a marked decrease in ALD patients (17.57±2.96mg/dL) when compared to healthy controls (23.73±4.94 mg/dL) and p value was found to be highly significant. Uric acid levels are markedly increased in ALD patients (6.60±1.32mg/dL) than controls (4.68±1.40 mg/dL) with a highly significant p value. Serum creatinine levels show an elevation of 1.12±0.55 mg/dL as compared to control subjects (0.88±0.16 mg/dL) and a significant p Value. Blood levels of conjugated bilirubin are noticeably higher in ALD patients 11. Similar results were achieved in this investigation, and Table 1(see PDF) indicates direct bilirubin levels of 2.03+ 3.05mg/dL and 0.41+ 0.53mg/dL in patients and controls, respectively. In comparison to healthy controls, this rise is approximately four to five times higher in ALD. The p value was determined to be meaningful (p 0.01). Table 1(see PDF) displays total bilirubin values of 3.61+4.91 mg/dL in patients with alcoholic liver disease and 0.92+0.91 mg/dL in controls, with a significant difference of p = 0.004 between the two groups.

## Discussion:

The rate at which proteins are catabolized in the liver is slowed down by alcohol consumption. In this study, table 1 demonstrates a decrease in the mean serum total protein content of 5.97 + 0.99 g/dL in ALD cases compared to 6.88 + 0.56 g/dL in healthy controls, with a very significant p value (0.001). In cases of ALD, which is frequently accompanied by malnutrition, albumin synthesis declines [12-13] As shown in Table 1(see PDF), this study also reveals that the mean albumin concentration in cases is 3.27+0.89g/dL, which is lower than the 4.28+0.39g/dL of healthy controls. The Royal College of Physicians issued a recommendation and suggested a safe weekly intake limit of 21 units, or around 168 g of alcohol [14]. However, frequent and regular ingestion damages multiple organs. The liver is frequently impacted by alcoholism, but because it has a huge reserve capacity, the patient is often determined to be healthy despite this. Biochemical markers fluctuate as liver damage worsens, and the patient exhibits a clinical picture. The liver sustains significant damage as a result of alcoholism because it is the primary site where alcohol metabolism takes place [15]. On the basis of morphology, there are three basic categories of liver disease: parenchymal, hepato biliary, and vascular. Alcohol acts as a cytotoxic, harming the liver parenchyma. Alcoholic fatty liver, hepatitis, and ultimately cirrhosis are pathological manifestations of alcoholism-related liver disease. The first and most important thing that happens after chronic alcohol use causes fibrosis and then scar development is collagen accumulation in the liver. Research on the pathogenic processes leading to cirrhosis from fibrosis is ongoing [16]. For diagnosing individuals with alcoholic liver disease, ultrasound performed better than liver biopsy. Decompensated liver disease had a detection rate of 91.7 percent, compared to 81.48 percent for compensated liver disease [17].

Lipids are the most significant component of biological membranes. Long chain fatty acids are esterified in the liver, producing VLDL triglycerides. In the heart, muscle, and adipose tissue, lipoprotein lipase hydrolyzes these VLDL triglycerides and chylomicrons. By receptor-mediated endocytosis, the liver excretes nearly 70% of this component [18, 19]. The majority of endogenous cholesterol production occurs in the liver. Plasma lipoprotein levels are out of whack as a result of the liver damage caused by persistent alcohol use. Cirrhosis brought on by alcohol causes a drop in blood cholesterol, HDL, and LDL levels [20]. Presence of renal dysfunction in alcoholic liver disease indicates poor prognosis. Intrinsic renal diseases are common in ALD patients. Morbidity and mortality increases when alcoholic liver disease occurs along with renal impairment [21].Primary arterial vasodilation in splanchnic circulation leads to a decrease in systemic vascular resistance. When portal hypertension occurs in ALD along with these changes, renal dysfunctions will manifest [22]. The distribution of alcohol depends on blood flow through vascular organs like the brain, which rapidly balance plasma levels. HDL levels will rise and apo-A1 production will increase. The activity of the cholesterol ester transfer protein (CETP), which transports cholesterol from tissues to HDL, will be increased. Peripheral lipolysis is increased by alcohol consumption. Triacylglycerol will accumulate as fatty acid production is enhanced. As a result of the microsomal triglyceride transfer protein being inhibited, the liver's ability to export VLDL is reduced [23]. Alcohol use has varying effects on the human body depending on whether it is acute or chronic. Stroke embolics, traffic fatalities, and alcohol poisoning are all caused by acute alcoholism. Chronic drinking causes hyper uricemia, pancreatic inflammation, liver damage, and central nervous system disease [24]. A number of studies have demonstrated a linear association between ethanol use and liver disease [25]. Ethanol increases NADH levels and inhibits gluconeogenesis, which decreases the amount of glucose available and results in hypoglycemia. A massive buildup of fat in hepatic cells causes fatty liver due to increased lactate production and decreased fatty acid oxidation in the Krebs cycle [26].

## Conclusions:

Data shows that serum total cholesterol and HDL showed a linear decrease when the severity of ALD progresses from compensated to decompensated state. Serum uric acid showed an early increase in compensated stage of ALD. This study inferred that Total cholesterol, TGL, HDL and uric acid can be used both in classification criteria and early parameter for assessing the severity of progression of ALD.
